# Impact of Nurse Practitioner Practice Regulations on Rural Population Health Outcomes

**DOI:** 10.3390/healthcare6020065

**Published:** 2018-06-15

**Authors:** Judith Ortiz, Richard Hofler, Angeline Bushy, Yi-ling Lin, Ahmad Khanijahani, Andrea Bitney

**Affiliations:** 1 College of Health and Public Affairs, University of Central Florida, Orlando, FL 32816, USA; YLLin@knights.ucf.edu (Y.L.); khanijahani@knights.ucf.edu (A.K.); 2 College of Business Administration, University of Central Florida, Orlando, FL 32816, USA; Richard.Hofler@ucf.edu; 3 College of Nursing, University of Central Florida, Orlando, FL 32816, USA; Angeline.Bushy@ucf.edu; 4 College of Sciences, University of Central Florida, Orlando, FL 32816, USA; andrea.bitney@cru.org

**Keywords:** nurse practitioners, rural, scope of practice, patient outcomes, rural workforce

## Abstract

*Background:* For decades, U.S. rural areas have experienced shortages of primary care providers. Nurse practitioners (NPs) are helping to reduce that shortage. However, NP scope of practice regulations vary from state-to-state ranging from autonomous practice to direct physician oversight. The purpose of this study was to determine if clinical outcomes of older rural adult patients vary by the level of practice autonomy that states grant to NPs. *Methods:* This cross-sectional study analyzed data from a sample of Rural Health Clinics (RHCs) (n = 503) located in eight Southeastern states. Independent *t*-tests were performed for each of five variables to compare patient outcomes of the experimental RHCs (those in “reduced practice” states) to those of the control RHCs (in “restricted practice” states). *Results:* After matching, no statistically significant difference was found in patient outcomes for RHCs in reduced practice states compared to those in restricted practice states. Yet, expanded scope of practice may improve provider supply, healthcare access and utilization, and quality of care (Martsolf et al., 2016). *Conclusions:* Although this study found no significant relationship between Advanced Registered Nurse Practitioner (ARNP) scope of practice and select patient outcome variables, there are strong indications that the quality of patient outcomes is not reduced when the scope of practice is expanded.

## 1. Introduction

For decades, rural areas across the U.S. have experienced persistent shortages of primary care providers, leaving rural residents at greater risk for health problems and illness complications. Rural communities differ from each other; however, both rural and urban areas are becoming more culturally diverse. The Hispanic population, for example, as the fastest growing ethnic group in the U.S. [1,2] has dispersed across the 50 states to both rural and urban communities. Hispanics/Latinos, African Americans, and other subgroups differ from the majority population regarding their beliefs and preferences about health, illness, and their ability to access health care. These distinctions, in the absence of culturally sensitive healthcare, may contribute to health disparities of the subgroup as compared to the majority population. 

Nurses make up the largest segment of the health care profession. They work in a wide variety of settings and provide care to diverse populations. As the healthcare system undergoes transformation due in part to the Affordable Care Act (ACA), the nursing profession has a significant role in delivering quality, patient-centered, accessible, and affordable care. The Institute of Medicine’s (IOM’s) [3] report entitled “The Future of Nursing: Leading Change, Advancing Health” made recommendations pertaining to the roles of nurses in the changing healthcare landscape.

Of relevance to rural areas are the IOM’s recommendations for Removing Barriers to Practice and Care [4,5]. That is to say, if Advanced Practice Registered Nurses (including nurse practitioners) are permitted to practice to the full extent of their education and training, this could build the necessary workforce to satisfy the health care needs of an increasing number of people, especially those living in medically underserved regions. (Historically, in the literature the title nurse practitioner [NP] was used. Recently, the designated title is Advanced Registered Nurse Practitioner [ARNP]; hence, ARNP is used in this article.). 

While steps have been taken at both federal and state levels to relax ARNP practice restrictions, many states inhibit ARNPs’ ability to practice at the level for which they have been prepared. The IOM states: “Collaborative models of practice, in which all health professionals practice to the full extent of their education and training, optimize the efficiency and quality of care for patients and enhance the satisfaction of healthcare providers” [3] (p. 3). To facilitate formation of these models, and increase the accessibility of quality health care, the IOM recommends that the nursing community, other health professions groups, and policy makers, establish a common ground to remove scope of practice restrictions and increase interprofessional collaboration. 

Relaxing scope of practice restrictions could help ARNPs meet the critical demand for primary care services in rural (and urban) areas. However, regulations vary from state-to-state regarding ARNPs’ scope of practice and level of professional autonomy. Currently, the 50 states are categorized into the following three groups based on ARNP’s practice regulations [6,7].

Full Practice: State practice and licensure laws provides for all nurse practitioners to evaluate patients, diagnose, order and interpret diagnostic tests, initiate and manage treatments—including prescribing medications and controlled substances—under the exclusive licensure authority of the state board of nursing. This is the model recommended by the National Academy of Medicine (formerly the IOM) and National Council of State Boards of Nursing. 

Reduced Practice: State practice and licensure laws reduces the ability of nurse practitioners to engage in at least one element of ARNP practice. State law requires a career-long regulated collaborative agreement with another health provider in order for the ARNP to provide patient care; or, limits the setting of one or more elements of ARNP practice.

Restricted Practice: State practice and licensure laws restricts the ability of a nurse practitioner to engage in at least one element of NP practice. State law requires career-long supervision, delegation, or team-management by another health provider in order for the ARNP to provide patient care.

Research studies indicate that less restrictive ARNP scope of practice regulations can increase primary care access and utilization. In one study of persons residing in the full-practice states, 62% had higher geographic accessibility to primary care clinicians (including ARNPs), compared to only 35% in restricted practice states [8]. Furthermore, ARNPs’ practice and prescriptive independence had a positive impact on physician referral, health education and counseling services, and the number of medications taken by patients [9]. These recent findings confirm Sakr’s [10] early findings that ARNPs not only provided proper care of minor injuries, but performed better than ‘novice” physicians at reading medical history records and planning patient follow-up care.

Several studies have examined outcomes of patients treated by ARNPs as compared to those treated by physicians. A systematic review found no differences in patient outcomes (as measured by emergency department (ED) or urgent care visits, rehospitalization rates, and mortality rates) of patients treated by ARNPs compared to those treated by medical doctors (MDs) [11]. However, there is a lack of consensus about the effects of ARNP practice regulatory policies on population health outcomes [12]. 

The current study concerned persons residing in rural areas of the U.S., many of whom are “vulnerable” to poor health, in particular older adults, low resource, and persons of minority groups. The principal aim of the study was to determine how clinical outcomes of older adult patients vary by level of practice autonomy that states grant to ARNPs.

## 2. Materials and Methods

### 2.1. Study Design

This cross-sectional study included data from Rural Health Clinics (RHCs) providing health services in HHS Region 4 during 2013. Since the mid-1970s, ARNPs have played a significant role in the nation’s RHCs. These approximately 4000 Medicare-certified clinics are intended to address the inadequate supply of physicians serving rural Medicare patients, and to increase the use of non-physician providers [13]. The RHC was the unit of analysis throughout the study. The study was approved by the Institutional Review Board of the University of Central Florida (IRB00001138). 

The eight states that compose HHS Region 4 (Alabama, Florida, Georgia, Kentucky, Mississippi, South Carolina, Tennessee) have varying regulations regarding the scope of practice for ARNPs. By 2013, among these eight study states, three (AL, KY, and MS) had adopted reduced ARNP practice laws, and five states (FL, GA, NC, SC, and TN) had adopted restricted ARNP practice laws [8]. None had adopted full practice laws.

### 2.2. Data Sources

The secondary data used in this study included the Provider of Services (POS) files, Medicare beneficiaries’ inpatient and outpatient claims, outpatient revenue center files, and the Centers for Medicare and Medicaid Services (CMS) Cost Reports for RHCs. Variables for each RHC’s type (provider-based or independent) and location were constructed using data from the POS files. Patient outcome variables were constructed using the 2013 inpatient and outpatient claims and outpatient revenue center files. Finally, several RHC organizational characteristic variables were constructed using data from the 2013 CMS Cost Reports. 

### 2.3. Variables

#### 2.3.1. Outcome (Dependent) Variables

Five different patient outcomes were measured in this study: the 30-day readmission rate, and risk-adjusted rates of Ambulatory Care Sensitive Conditions for four principal diagnoses: COPD/Asthma, Congestive Heart Failure (CHF), Diabetes, and Pneumonia. The risk-adjustment statistical process controls for the potential effects of patient’s gender, age, race, comorbidity (using the Charlson Comorbidity Index), and the claim year. All of the variables were analyzed and reported on the RHC level. 

#### 2.3.2. Independent Variables

The primary independent variable was the geographic “location” of the RHC. “Location” was defined as whether the RHC were located in a state with reduced (the experimental group) or restricted (the control group) ARNP scope of practice laws. Also included in the analysis were control variables for each RHC’s characteristics, including the type of the RHC (independent or provider-based), “size” (measured as the total of physician + ARNP + PA FTEs), the percentage of ARNP FTEs (calculated as the number of ARNP FTEs divided by the “size”), and “rurality” (a measure of the RHC’s geographic location.) RHCs were categorized by degrees of “rurality” using the Rural-Urban Commuting Area Code, or RUCA [14]. This method categorizes the rural status of each RHC’s location based on Zip code. 

### 2.4. Analysis

We constructed a panel of clinics continuously certified as Region 4 RHCs from 2007 through 2013 using the Provider of Services (POS) file. This process resulted in a panel of 503 RHCs. During the study period, an average of approximately 463 patients were assigned to each RHC in the study panel. In that patients often visit more than one medical facility, patients were assigned to the RHC that provided the plurality of his or her services during the year 2013. The patient outcome variables from the Medicare beneficiaries’ claims were aggregated to the clinic level. 

The study aimed to measure the effects of state regulatory laws regarding ARNP scope of practice on health outcomes. We attempted to eliminate (or at least, minimize) the effects of care provided by other clinicians with similar levels of authority—physicians and physician assistants of the RHCs. For this reason, only RHCs in which ARNPs accounted for 90% or more of a clinic’s total FTEs (full-time equivalents) were included in the analysis. Of the 503 RHCs in the study panel, we found 77 where ARNPs made up 90% or more of their professional clinical staff. 

Among the 77 RHCs with 90% of more ARNPs, two groups were created: RHCs in reduced practice states (the experimental group), and RHCs in restricted practice states (the comparison group.) The comparison group was constructed using the statistical technique “propensity score matching” (PSM) described in the next section.

#### 2.4.1. Comparison Sample Construction 

Propensity score matching (PSM) is used with secondary data to control for bias in the assignment to the experimental and control groups [15]. Variable selection for classic propensity score models consider only the exposure (for our study, RHCs in either reduced or restricted practice states). However, according to one study, variables unrelated to the exposure but related to outcomes can be included in a PSM model to increase the precision of the estimated exposure effect without increasing bias [16]. Thus, for the current study, variable selection for the propensity score models included three variables for RHC characteristics that are related to patient outcomes: “size,” “rurality,” and “type.”

PSM was performed separately for each of the five patient outcome variables using radius matching. In each model, based on the structure of data, the specific caliper width was defined. To ensure good match quality, a caliper of one-fourth the standard deviation of each clinic’s propensity scores was used, and bias balancing diagnostics such as standardized biased, variance ratio tests, *t*-tests, and Rubin’s B and R statistics were evaluated.

#### 2.4.2. Treatment of Missing Data

Some of the missing values in 2013 for variables in the propensity score models and patient outcomes were replaced using mean or linear estimation using the available data for the same variables in at least three years (from 2007–2012, and 2014). Since the number of study cases was limited, it was assumed that these replaced missing values could add to the quality of matching and further analysis by increasing the number of the valid observations (values). Table 1 shows the missing imputation method approaches and the imputation quality. We found no substantial differences in the means and standard deviations of the imputed variables before and after the imputation methods were applied. 

#### 2.4.3. Independent *t*-Test

While performing the PSM technique, we controlled for three RHC-related variables: size, type, and “rurality.” Since the effects of these factors were controlled for in the matching process, independent *t*-tests were then performed for each of the five outcome variables to compare the outcomes of the experimental RHCs (those in reduced practice states) to those of the control RHCs (those in restricted practice states).

## 3. Results

Table 2 shows characteristics of the experimental and control RHCs. Bivariate analysis of these characteristics was conducted, but no statistically significant differences were observed. Of the 77 RHCs, 32 (41.56%) were located in states with restricted ARNP practice law; and, 45 (58.44%) were in states with reduced ARNP practice law. More than 70% of the total RHCs were independent; less than 30% of them were provider-based. Most (34.4%) of the RHCs in restricted practice states belonged to RUCA category “urban,” whereas more than half of the RHCs in reduced practice states were classified into RUCA categories “large” and “small” rural. The mean RHC “size” in restricted practice states was 1.42 FTEs, whereas for those in reduced practice states it was 1.52. 

Table 3 shows the results of the bivariate analyses for the five patient outcomes. After matching, no statistically significant difference was found in patient outcomes for RHCs in the restricted practice states as compared to those in reduced practice states. 

## 4. Discussion

Nurse practitioners contribute to U.S. healthcare by providing health services individually and as a part of healthcare teams that include physicians, nurses and other health care professionals. Recruiting ARNPs and expanding their scope of practice to include providing diagnoses, prescriptions, treatments, consultations and other services could shift some of healthcare burden away from physicians. ARNPs functioning at the level for which they are prepared could help to improve healthcare access disparities in areas with severe physician shortages such as rural areas, where shortages have persisted and are anticipated for the foreseeable future.

This study compared healthcare outcomes of Medicare beneficiaries of RHCs located in states with reduced ARNP scope of practice laws to those with restricted laws. After controlling for individual patient-level confounders (using risk adjustment procedures), and RHC characteristics, no significant difference was observed between the two groups of RHCs for any of five patient outcomes. However, it is important to stress that populations derive other benefits when ARNP scope of practice is less restrictive.

According to the findings of a recent study by Graves and colleagues [8], restrictions on ARNPs’ scope of practice were associated with up to 40% fewer primary care nurse practitioners (PCARNPs) in states with restricted ARNP practice environment compared to their full practice counterparts. These practice restrictions may contribute to limitations in access to primary healthcare services and perpetuate rural health disparities. Relaxing restrictions on ARNP scope of practice may also expand the capacity of primary care services in rural areas. Based on a review of 14 published articles focusing on ARNP scope of practice and its effect on quality of care, cost, health care utilization and outcomes, Martsolf and colleagues [17] suggest that expanded scope of practice improved the provider supply, healthcare access and utilization, and quality of care.

It is important to stress that professional and personal satisfaction is ideal for many ARNPs in rural practice, especially for those who have rural life experiences (i.e., having been raised, lived, or spent time in a rural setting) [18]. However, it is also important to note that a dichotomy exists in states having large rural and remote areas as well as less restrictive regulations regarding ARNP scope of practice. Specifically, many rural communities, particularly those in isolated regions, have the least restrictive APRN scope of practice regulations. Yet, these regions experience serious, consistent challenges in recruiting and retaining health professionals of all types—physicians, mental and behavioral health professionals, as well as ARNPs [19]. 

Several reasons are offered to explain why APRNs may choose ***not*** to practice in a state with the least restrictive scope of practice. Among others, there is an urban bias in the higher education system where professional clinical experiences tend to take place in an urban-based healthcare facility. Urban-based education contributes to minimal (if any) student exposure to rural populations, cultural preferences and practice environments. Another contributing factor is lower reimbursement by third party payers for primary care services coupled with disparate reimbursement for APRNs as well as rural healthcare providers. 

Low population density in rural regions often can support only one or two providers; which, in turn, may lead to a sense of professional isolation for the ARNP (or physician) who chooses to practice in these more remote sites. This reality is often compounded by an expectation that the ARNP be available or “on call 24/7” to see patients with minimal backup support. Such professional community expectations and needs often conflict with an ARNP’s personal and family roles. All too often, the result is a decision to relocate to a practice setting that better suits the ARNP’s personal, professional and family preferences. 

The findings of the current study should be viewed considering a few limitations. First, attributing changes in patient outcomes solely to the RHC’s services may give an incomplete picture. The change in health status of a patient is a result of services provided by a group of healthcare providers, including ARNPs. Even after including only RHCs with 90% or more ARNP staff, the impact of care given by other healthcare providers such as physicians should not be ignored. Second, there is considerable variation in ARNP scope of practice among states within a scope of practice category (i.e., reduced, restricted). For example, one state may only restrict the ARNPs in prescribing medicine, but allow full authority to diagnose, treat, and consult patients, whereas another state may restrict ARNPs in all aspects of their practice. Finally, this study was limited to one year of data.

## 5. Conclusions 

Although this study found no significant relationship between ARNP scope of practice and select patient outcome variables, there are strong indications that the quality of patient outcomes is not reduced when the scope of practice is expanded. Well-qualified and high-performing ARNPs may positively contribute to access and utilization of primary care services in rural areas (Institute of Medicine, 2010). The increased access and reduced costs may warrant expanded nurse practitioner autonomy, particularly in rural and other underserved areas. Further longitudinal research that includes additional patient outcome indicators may broaden our understanding of the patient experience with nurse practitioner services, as well as the relationship between their scope of practice and patient outcomes.

## Figures and Tables

**Table 1:** Frequency, Mean, and Standard Deviation before and after Missing Value Treatment.

Variable	Missing Imputation Method	Number of Valid Cases		Mean		Std. Deviation	
		Before	After	Before	After	Before	After
RHC Size	Mean	58	77	1.5278	1.4764	1.0533	0.95711
% NP FTEs	Mean	58	77	0.9859	0.9856	0.02847	0.02927
R_Readm13	Linear Regression	68	74	0.1760	0.1725	0.01971	0.02561
R_ACSCCOPD_Rate13	Linear Regression	63	72	0.0651	0.0631	0.03242	0.031
R_ACSCDiab_Rate13	Linear Regression	41	61	0.0293	0.0261	0.01916	0.01744
R_ACSCHF_Rate13	Linear Regression	50	65	0.1346	0.1271	0.04343	0.04149
R_ACSCPneu_Rate213	Mean	68	74	0.0093	0.0083	0.00359	0.00294

**Table 2:** Rural Health Clinic(RHC) Characteristics by Nurse Practitioner State Practice in Region 4

Characteristic	State with Restricted Practice (n = 32)		State with Reduced Practice (n = 45)		*p*-Value
	Mean/n	SD/%	Mean/n	SD/%	
RHC Type					0.196
Independent RHC	25	78.1%	29	64.4%	
Provided-Based RHC	7	21.9%	16	35.6%	
RUCA * Category					0.545
Urban	11	34.4%	9	20.0%	
Large Rural	7	21.9%	13	28.9%	
Small Rural	8	25.0%	12	26.7%	
Isolated	6	18.8%	11	24.4%	
RHC Size (No. of Physician + PA + NP FTEs)	1.42	0.81	1.52	1.05	0.649

* The Rural-Urban Commuting Area (RUCA) Code has four categories: 1 = Urban, 2 = Large rural, 3 = Small rural, and 4 = Isolated. This binary variable “rural” equals one for each RHC with a RUCA code equal to 4. We chose this specification for this binary variable because our analysis of survey responses we received showed that RHCs in isolated areas are less likely to join ACOs.

**Table 3:** Effect of the State’s NP Practice Law on Patient Outcomes in 2013 in Region 4 Rural Health Clinic Using Independent *t*-test.

Patient Outcomes	State with Restricted Practice			State with Reduced Practice			*p*-Value
	n	Mean	SD	n	Mean	SD	
Readmission rate within 30 days	31	16.69%	2.64%	39	17.65%	2.51%	0.126
Hospitalization rate for COPD or Asthma	30	6.17%	3.68%	33	6.38%	2.91%	0.804
Hospitalization rate for Diabetes	25	2.24%	1.09%	28	2.68%	2.16%	0.349
Hospitalization rate for Heart Failure	27	12.48%	3.94%	29	13.31%	4.48%	0.467
Hospitalization rate for Pneumonia	31	0.86%	0.30%	39	0.79%	0.26%	0.303
